# The role of remnant cholesterol beyond low-density lipoprotein cholesterol in diabetes mellitus

**DOI:** 10.1186/s12933-022-01554-0

**Published:** 2022-06-27

**Authors:** Xiangming Hu, Qunzhi Liu, Xingyuan Guo, Weimian Wang, Bingyan Yu, Beijia Liang, Yingling Zhou, Haojian Dong, Jijin Lin

**Affiliations:** 1 Department of Cardiology, Guangdong Provincial Key Laboratory of Coronary Heart Disease Prevention, Guangdong Provincial People’s Hospital, Guangdong Cardiovascular Institute, Guangdong Academy of Medical Sciences, Guangzhou, 510080 Guangdong China; 2 The Second School of Clinical Medicine, Southern Medical University, Guangzhou, 510515 Guangdong China; 3 Shantou University Medical College, Shantou, 515041 Guangdong China

**Keywords:** Low-density lipoprotein cholesterol, Remnant cholesterol, Diabetes mellitus, Discordance, Concordance, Inflammation, Insulin resistance

## Abstract

**Background:**

Previous research has linked elevated low-density lipoprotein cholesterol (LDL-C) and remnant cholesterol (RC) with diabetes mellitus (DM). The present study aims to estimate the RC-related DM risk beyond LDL-C, and to investigate the extent to which the association of RC and DM is mediated via insulin resistance and inflammation.

**Methods:**

We enrolled 7308 individuals without previous history of DM into the present study from the China Health and Nutrition Survey. Fasting RC was calculated as total cholesterol minus LDL-C and high-density lipoprotein cholesterol. Subjects were divided into four groups according to their LDL-C (100 mg/dL) and RC (24 mg/dL) levels to evaluate the role of LDL-C vs. RC on DM. A logistic regression analysis was then employed to evaluate the relationships between the discordant/concordant LDL-C and RC and DM. A mediation analysis was undertaken to identify potential mediators.

**Results:**

Of all the participants, a total of 625 (8.55%) patients were newly diagnosed with DM. Compared to the high LDL-C/low RC group, the low LDL-C/high RC group was more common in DM patients. After a multivariate adjustment, elevated LDL-C and RC were associated with DM. Moreover, the low LDL-C/high RC group and the high LDL-C/low RC group manifested a 4.04-fold (95% CI 2.93–5.56) and 1.61-fold (95% CI 1.21–2.15) higher risk of DM, relative to those with low LDL-C/low RC. The subgroup analysis indicated that low LDL-C/high RC was more likely to be related to DM in females. Similar results were also shown when the sensitivity analyses were performed with different clinical cut-points of LDL-C. Insulin resistance and inflammation partially mediated the association between RC and DM.

**Conclusions:**

Our findings provided evidence for RC beyond the LDL-C associations with DM that may be mediated via insulin resistance and the pro-inflammatory state. In addition, women are more susceptible to RC exposure-related DM.

**Supplementary Information:**

The online version contains supplementary material available at 10.1186/s12933-022-01554-0.

## Background

Diabetes mellitus (DM) is a metabolic disorder that is characterized by persistent hyperglycemia due to defects in glucose metabolism. According to the International Diabetes Federation, 536.6 million people worldwide aged 21–79 were afflicted with DM in 2021 [1]. With rapid developments in the global economy and changes in diet, the high morbidity and disability exhibited in DM has become a significant public health issue that has reached alarming levels.

Previous studies have shown that dyslipidemia appears in the early stages of DM and continues to be involved in the disease progresses throughout its entire course [2–4]. Among the spectrum of lipoproteins, there is no doubt that low-density lipoprotein cholesterol (LDL-C) is the most important one. However, with the widespread use of statins in recent years, we have shifted our attention to triglyceride (TG)-rich lipoproteins (TRLs), one of the hallmarks of diabetic dyslipidemia that cannot be controlled by statins [5, 6]. There has been increasing evidence in recent years that TRLs are associated with the onset of diabetes [7]. A molecular lipid analysis showed that plasma TG levels were the only variable associated with DM in high-risk groups [8], and a high-fat diet increased the synthesis of TG in the liver, reduced relative high-density lipoprotein cholesterol (HDL-C), and accelerated the onset of DM [9]. In population cohort studies, a higher TG-related indicators were associated with DM independent of insulin resistance, highlighting the importance of TRLs in the development of DM [10–12].

Remnant cholesterol (RC) is the cholesterol content of TRLs that consists of very low-density lipoproteins (VLDL), intermediate-density lipoproteins (IDL), and chylomicron remnants [13]. Although plasma TG can be used as a surrogate measure of TRLs or RC clinically, they represent different lipid disorders. In fact, RC is essentially a cholesterol nature. Cholesterol content in TRLs contributed more directly to cardiovascular disease (CVD) rather than TG, thus, RC was highlighted in the lipids management in recent years [14]. RC is more abundant, larger, and carries more cholesterol than LDL-C particles, so it can be inferred that RC is more harmful to pancreatic β-cells [8]; In addition, unlike LDL-C, TRLs exert their pathogenic effects through inflammatory pathways [15, 16]. Epidemiological evidence has suggested that higher RC levels were not only significantly associated with the development of DM, but also contributed to the comorbidities such as hypertension [17, 18]. In recent years, high levels of RC have been proposed as part of the explanation for the residual risk after optimal LDL-C was proposed as a viewpoint in primary prevention [19, 20]. Interestingly, as research continues, it appears that the association between RC and CVD extends beyond LDL-C [21, 22], introducing a new point of the discordant/concordant LDL-C and RC. It will become increasingly important to investigate the discordance/concordance of LDL-C and RC, especially in the context of the increasing prevalence of DM, as both are on the rise in DM patients. Specific TRLs and LDL-C particles have been linked to DM based on the Prevention of Renal and Vascular End-Stage Disease (PREVEND) study [8], but the association between the discordant/concordant LDL-C and RC and DM requires further elucidation.

The prevalence of DM in China ranks first among the world’s countries and continues to exhibit an annual upward trend [1, 23] that is closely related to the rapid development of the economy and the changes in the dietary structure. The prevalence of obesity and dyslipidemia increased significantly during this same period [24]. The disparate prognostic outcomes of the LDL-C and RC discordance/concordance prompt further exploration of the high-risk population and the potential mechanism for this phenomenon. In this study, we aimed to identify the discordant/concordant LDL-C and RC associations with DM and explore whether insulin resistance and inflammation mediate this relationship, based on the China Health and Nutrition Survey.

## Methods

### Study setting and population

The China Health and Nutrition Survey (CHNS) is an ongoing longitudinal community-based cohort study carried out by the national and local governments of China, and the study details were described in our previous article [25]with the relevant protocol published elsewhere [26]. The CHNS is a collaborative project between the Carolina Population Center, University of North Carolina at Chapel Hill and the National Institute for Nutrition and Health (NINH, former National Institute of Nutrition and Food Safety), Chinese Center for Disease Control and Prevention. The study protocols and ethics approval were derived from the Institutional Review Committees of the University of North Carolina at Chapel Hill and the National Institute for Nutrition and Health at Beijing and so, were attributed to the two centers. Each participant was enrolled in China and provided written informed consent.

In 2009, the CHNS collected 9549 fasting blood samples. We excluded 841 participants who were under 18 years, 62 pregnant women, 281 participants previously diagnosed with DM, and 89 who were previously diagnosed with myocardial infarction—thus avoiding the influence of possible lipid-lowering therapies. We also declined to enroll 917 patients who were missing RC information or whose RC was less than or equal to 0, and 51 patients who lacked information regarding their glycosylated hemoglobin A1c (HbA1c) or fasting blood glucose (FBG) with respect to the DM diagnosis. Our current study population eventually comprised 7308 individuals (Additional file 1: Fig. S1), and we listed the patient information that includes the baseline characteristics, healthy behaviors, and laboratory examinations.

### Measures

RC (mmol/L) was calculated from a standard lipid profile of the patient in a fasting state as total cholesterol (TC) (mmol/L) minus LDL-C (mmol/L) minus HDL-C (mmol/L) [27]. According to the American Diabetes Association criteria, DM was defined as exhibiting a previous DM history or a FBG ≥ 7.0 mmol/L and/or an HbA1c ≥ 6.5% [28].

### Definitions

Height and weight were measured while the subjects were wearing light clothing and standing without shoes. We calculated the body mass index (BMI) as weight (kg)/height (m)^2^. Health behaviors (smoking and alcohol consumption) were self-reported. Smoking was defined as any previous smoking (yes/no), and alcohol consumption was defined as imbibition greater than three times per week (yes/no).

Participants were asked to maintain a regular pattern of life as well as normal emotional stability for at least three days, and to fast for 8–12 h prior to blood sample collection. From the blood samples, the white blood cells (WBCs) was measured with a Beckman Coulter LH751 (Beckman Coulter, USA). The LDL-C, HDL-C, TC, TG, apolipoprotein B (apoB), apolipoprotein A (apoA), FBG, uric acid, and serum creatinine were measured using a Hitachi 7600 machine (Randox, UK and Kyowa, Japan). The HbA1c was determined by the HLC-723 G7/D10/PDQ A1c (Tosoh, Japan/Bio-Rad, USA/Primus, USA), and the high-sensitivity C-reactive protein (Hs-CRP) was detected with a Hitachi 7600 (Denka Seiken, Japan). Elevated Hs-CRP was indicated as ≥ 5 mg/L. We calculated the estimated glomerular filtration rate (eGFR) (mL/min 1.73 m^− 2^) employing the Chronic Kidney Disease—Epidemiology Collaboration (CKD-EPI) equation, and the CKD was diagnosed based on an eGFR of < 60 mL/min 1.73 m^− 2^. The homeostasis model assessment of insulin resistance (HOMA-IR) was calculated by the formula HOMA-IR = serum insulin (mU/mL) * FBG (mmol/L)/22.5 [29], while the homeostasis model assessment of insulin sensitivity (HOMA-IS) was calculated by the formula HOMA-IS = 1/HOMA-IR.

### Statistical analyses

To assess the impact of RC beyond the LDL-C in DM patients, we selected 2.6 mmol/L (100 mg/dL) and 0.62 mmol/L (24 mg/dL) as the cut-off points for the LDL-C and RC, respectively, according to the recommended LDL-C target from the 2019 ESC/EAS Guidelines for the Management of Dyslipidaemias [30] and a recent primary prevention study of RC [21]. Hence, we divided all the participants into four groups (Group 1: low LDL-C/low RC, Group 2: low LDL-C/high RC, Group 3: high LDL-C/low RC, and Group 4: high LDL-C/high RC).

Participant characteristics were described according to whether DM was present and commensurate with the discordance/concordance of LDL-C and RC. Continuous variables are expressed as means ± standard deviation for normal distributions or medians and interquartile range (25–75%) for skewed distributions, and categorical variables are presented as relative frequencies (percentages). We employed *t*-tests for normally distributed data and the Mann-Whitney U/Kruskal-Wallis rank test for non-normally distributed data; The Chi-squared test/Fisher’s exact-probability test for categorical variables was used to determine significant differences between the groups. The independent association of the discordance/concordance of the LDL-C and RC with the presented DM was evaluated using logistic models with odds ratios (ORs) and 95% confidence intervals (CIs). Potential covariates that were significant in the baseline comparison or that we considered to be of clinical importance were included in the multivariate models. We constructed four main models for the covariate adjustment, i.e., Model 1, unadjusted; Model 2, adjusted for age, sex, and BMI; and Model 3, adjusted for the variables in Model 1, educational level, smoking, alcohol consumption, and CKD. Since previous studies have reported that RC is closely related to TG, we adjusted for the TG in Model 4 for further validation. In addition, as previous anti-hypertensive drug use and the LDL-C size that can be reflected by the LDL-C/Apo-B ratio and are associated with the development of DM, we also adjusted for the use of anti-hypertensive drugs and the LDL-C/Apo-B ratio in Model 5.

The subgroup analysis was executed to examine the effect of the discordant/concordant LDL-C and RC on DM in the various subgroups, including age (</≥ 65 years) and sex (male/female). We tested the modifications and interactions of the subgroups using the likelihood-ratio test. For sensitivity analysis, using the same model, we assessed the relationship between the discordant/concordant RC and LDL-C groups and DM using a more stringent clinical cut-point for LDL-C (1.80 mmol/L).

Given that the pro-inflammatory state and insulin resistance have been identified as pathways through which DM may affect the metabolism of serum lipids [31, 32], we investigated whether the association between the discordant/concordant LDL-C and RC and DM was mediated by WBCs, hs-CRP, and HOMA-IR. The Sobel test was used to assess the effects of these mediators [33].

The proportion of missing data in the analytic sample was not greater than 2%, and the missing data were then interpolated using the method of the last observation carried forward, or using the means and medians for the continuous variables and skewed variables. Comparisons where P was < 0.05 (two-sided) were considered to be statistically significant. We performed all analyses with Stata 15.0, R (version 3.4.3) and EmpowerStats (http://www.empowerstats.com, X&Y Solutions, Inc., Boston, MA, USA).

## Results

### Baseline characteristics

**Fig. 1:**
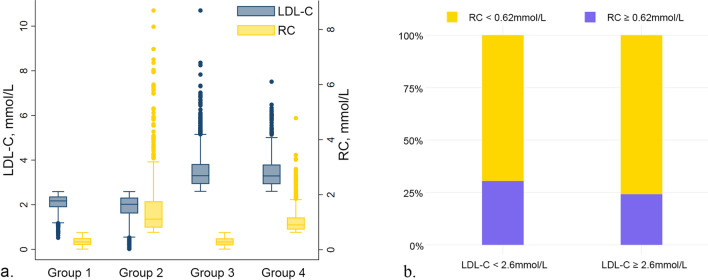
The distribution of LDL-C and RC by the discordant/concordant groups. **a** The box plot of the different LDL-C and RC levels.** b** the relative proportions of the discordant/concordant groups. Groups 1–4 represent the low LDL-C/low RC group, low LDL-C/high RC group, high LDL-C/low RC group and the high LDL-C/high RC group, respectively. *LDL-C* low-density lipoprotein cholesterol, *RC* remnant cholesterol

The distribution of LDL-C and RC by the discordant/concordant groups is shown in Fig. 1. A high level of RC accounted for a relatively small proportion of the participants with low or high levels of LDL-C, while higher RC was more distributed in the low LDL-C group. Baseline characteristics of participants according to the discordance/concordance of the LDL-C and RC levels are shown in Table 1. Compared with patients in Group 3 (high LDL-C/low RC), those in Group 2 (low LDL-C/high RC) tended to be younger, were males, smokers and drinkers, and had higher BMI and blood pressure. The prevalence of DM was higher in Group 2 (15.93%) than in Group 3 (7.51%). As for the cardiometabolic biomarkers, Group 2 had lower levels of HDL-C TC and lipoprotein (a) than Group 3, while TG in Group 2 is lower than that in Group 3. Group 2 was also accompanied by an increase in the HOMA-IR, WBCs, and hs-CRP. Baseline information of our enrolled participants by DM and non-DM groups was also summarized in Table 2, exhibiting significantly higher LDL-C and RC levels in DM compared with the control group. DM patients tended to be older, and also tended to possess a higher BMI, blood pressure, WBC count, and hs-CRP. In addition, they manifested lower HDL-C and lipoprotein (a). For the discordance/concordance of LDL-C and RC, Group 2 (low LDL-C/high RC) and Group 4 (high LDL-C/high RC) were more common in DM patients.

**Table 1:** Baseline characteristics of enrolled participants among groups of discordant/concordant LDL-C and RC

	Group 1 low LDL-C and low RC n = 1921	Group 2 low LDL-C and high RC n = 841	Group 3 high LDL-C and low RC n = 3448	Group 4 high LDL-C and high RC n = 1098	p-value^†^
Age, years	45 ± 16	48 ± 14	52 ± 14	52 ± 14	< 0.001
BMI, kg/m^2a^	21.97 ± 3.08	24.36 ± 3.55	23.38 ± 3.31	25.15 ± 3.44	< 0.001
Male sex	879 (45.76%)	488 (58.03%)	1548 (44.90%)	563 (51.28%)	< 0.001
Junior high school or above	1149 (59.81%)	486 (57.79%)	1894 (54.93%)	629 (57.29%)	0.006
Residence					0.208
Urban	583 (30.35%)	284 (33.77%)	1109 (32.16%)	366 (33.33%)	
Rural	1338 (69.65%)	557 (66.23%)	2339 (67.84%)	732 (66.67%)	
Currently smoking	631 (32.85%)	364 (43.28%)	1185 (34.37%)	443 (40.35%)	< 0.001
Alcohol consumption	215 (11.19%)	162 (19.26%)	451 (13.08%)	143 (13.02%)	< 0.001
Chronic kidney disease	138 (7.18%)	67 (7.97%)	467 (13.54%)	151 (13.75%)	< 0.001
Hypertension	134 (6.98%)	115 (13.67%)	398 (11.54%)	206 (18.76%)	< 0.001
DM	65 (3.38%)	134 (15.93%)	259 (7.51%)	167 (15.21%)	< 0.001
LDL-C, mmol/L	2.10 ± 0.36	1.90 ± 0.53	3.47 ± 0.72	3.44 ± 0.70	< 0.001
HDL-C, mmol/L	1.48 ± 0.37	1.11 ± 0.34	1.48 ± 0.33	1.21 ± 0.25	< 0.001
TG, mmol/L	0.99 ± 0.48	3.72 ± 2.61	1.30 ± 0.70	2.91 ± 1.29	< 0.001
Total cholesterol, mmol/L	3.86 ± 0.50	4.48 ± 0.89	5.22 ± 0.80	5.68 ± 0.85	< 0.001
RC, mmol/L	0.27 (0.16–0.41)	1.10 (0.79–1.75)	0.27 (0.15–0.40)	0.89 (0.72–1.16)	< 0.001
Apo-B, g/L	0.64 ± 0.13	0.78 ± 0.17	1.00 ± 0.22	1.16 ± 0.23	< 0.001
Apo-A, g/L	1.14 ± 0.36	1.06 ± 0.45	1.17 ± 0.39	1.11 ± 0.31	< 0.001
LDL-C/Apo-B	1.33 ± 2.20	1.00 ± 1.42	1.41 ± 2.52	1.16 ± 0.13	< 0.001
Lipoprotein (a), mg/L	64.00 (33.00-131.00)	49.00 (23.00–97.00)	95.00 (50.00-199.25)	77.00 (42.00-170.00)	< 0.001
Uric acid, µmol/L^a^	275.93 ± 81.70	394.00 ± 171.96	296.05 ± 83.72	358.88 ± 94.96	< 0.001
WBC count, 10^9^/L^a^	6.12 ± 2.00	6.67 ± 2.58	6.25 ± 1.86	6.75 ± 1.82	< 0.001
Hs-CRP, mg/L^a^	1.00 (0.00–2.00)	1.00 (1.00–3.00)	1.00 (1.00–2.00)	2.00 (1.00–3.00)	< 0.001
Serum creatine, µmol/L^a^	85.21 ± 32.50	86.80 ± 15.46	88.21 ± 17.27	90.13 ± 16.35	< 0.001
HbA1c, %	5.37 ± 0.56	5.65 ± 0.95	5.60 ± 0.73	5.77 ± 0.94	< 0.001
FBG, mmol/L	4.97 ± 0.81	5.77 ± 1.75	5.29 ± 1.09	5.75 ± 1.71	< 0.001
Insulin, uIU/mL	9.32 (6.69–13.03)	12.06 (8.14–19.63)	10.28 (7.29–14.31)	13.72 (9.52–20.21)	< 0.001
HOMA-IR^a^	2.00 (1.39–2.90)	2.93 (1.84–5.04)	2.33 (1.61–3.40)	3.30 (2.21–5.13)	< 0.001
HOMA-IS^a^	0.50 (0.34–0.72)	0.34 (0.20–0.54)	0.43 (0.29–0.62)	0.31 (0.19–0.46)	< 0.001
Anti-hypertensive drugs	99 (5.15%)	93 (11.06%)	312 (9.05%)	164 (14.94%)	< 0.001

*BMI* body mass index, *DM* diabetes mellitus, *LDL-C* low-density lipoprotein cholesterol, *HDL-C* high-density lipoprotein cholesterol, *TG* triglyceride; *RC* remnant cholesterol, *Apo-A* apolipoprotein A, *Apo-B* apolipoprotein B, *WBC* white blood cell, *Hs-CRP* high-sensitivity C-reactive protein, *HbA1c* glycosylated hemoglobin A1c, *FBG* fasting blood glucose, *HOMA-IR* homeostasis model assessment of insulin resistance, *HOMA-IS* homeostasis model assessment of insulin sensitivity

^a^Mean ± SD, median (Q1-Q3), or n (%) was calculated after filling in missing data

^†^p-values by Kruskal-Wallis rank test

**Table 2:** Baseline characteristics of enrolled participants according to presence or absence of DM

RC	Non-DM n = 6683	DM n = 625	P value
Age, year	49.36 ± 15.04	57.92 ± 13.36	< 0.001
BMI, kg/m^2a^	23.22 ± 3.38	25.12 ± 3.83	< 0.001
Male sex	3162 (47.31%)	316 (50.56%)	0.120
Junior high school or above^a^	3879 (58.04%)	279 (44.64%)	< 0.001
Residence			0.950
Urban	2141 (32.04%)	201 (32.16%)	
Rural	4542 (67.96%)	424 (67.84%)	
Currently smoking	2380 (35.61%)	243 (38.88%)	0.103
Alcohol consumption	864 (12.93%)	107 (17.12%)	0.003
Hypertension	699 (10.46%)	154 (24.64%)	< 0.001
LDL-C, mmol/L	2.91 ± 0.90	3.08 ± 1.17	< 0.001
HDL-C, mmol/L	1.40 ± 0.36	1.29 ± 0.37	< 0.001
TG, mmol/L	1.63 ± 1.27	2.92 ± 2.68	< 0.001
Total cholesterol, mmol/L	4.80 ± 0.98	5.37 ± 1.14	< 0.001
RC, mmol/L	0.35 (0.19–0.61)	0.59 (0.32–1.20)	< 0.001
Apo-A, g/L	1.15 ± 0.39	1.10 ± 0.30	0.002
Apo-B, g/L	0.89 ± 0.26	1.02 ± 0.29	< 0.001
LDL-C/Apo-B	1.32 ± 2.22	1.15 ± 0.28	0.059
Lipoprotein (a), mg/L	77.00 (40.00-165.00)	74.00 (34.00-157.00)	0.053
Uric acid, µmol/L^a^	307.32 ± 99.32	355.87 ± 159.58	< 0.001
WBCs, 10^9^/L^a^	6.29 ± 1.95	6.91 ± 2.40	< 0.001
Hs-CRP, mg/L^a^	1.00 (0.00–2.00)	2.00 (1.00–4.00)	< 0.001
Serum creatine, µmol/L^a^	87.33 ± 21.88	89.83 ± 24.25	0.007
HbA1c, %	5.43 ± 0.45	7.04 ± 1.54	< 0.001
FBG, mmol/L	5.08 ± 0.62	7.95 ± 2.68	< 0.001
Insulin, uIU/mL	10.27 (7.29–14.55)	16.13 (10.21–27.50)	< 0.001
HOMA-IR^a^	2.28 (1.58–3.34)	5.59 (3.12–10.24)	< 0.001
HOMA-IS^a^	0.44 (0.30–0.63)	0.18 (0.10–0.32)	< 0.001
Discordance of LDL-C and RC			< 0.001
Low LDL-C/low RC	1856 (27.77%)	65 (10.40%)	
Low LDL-C/high RC	707 (10.58%)	134 (21.44%)	
High LDL-C/low RC	3189 (47.72%)	259 (41.44%)	
High LDL-C/high RC	931 (13.93%)	167 (26.72%)	
Anti-hypertensive drugs	546 (8.17%)	122 (19.52%)	< 0.001

*DM* diabetes mellitus, *BMI* body mass index, *LDL-C* low-density lipoprotein cholesterol, *HDL-C* high-density lipoprotein cholesterol, *TG* triglyceride, *RC* remnant cholesterol, *Apo-A* apolipoprotein A, *Apo-B* apolipoprotein B, *WBC* white blood cell, *Hs-CRP* high-sensitivity C-reactive protein, *HbA1c* glycosylated hemoglobin A1c, *FBG* fasting blood glucose, *HOMA-IR* homeostasis model assessment of insulin resistance, *HOMA-IS* homeostasis model assessment of insulin sensitivity

^a^Mean ± SD, median (Q1-Q3), or n (%) was calculated after filling in missing data

### Inter-relation of the discordant/concordant LDL-C and RC and DM

**Fig. 2:**
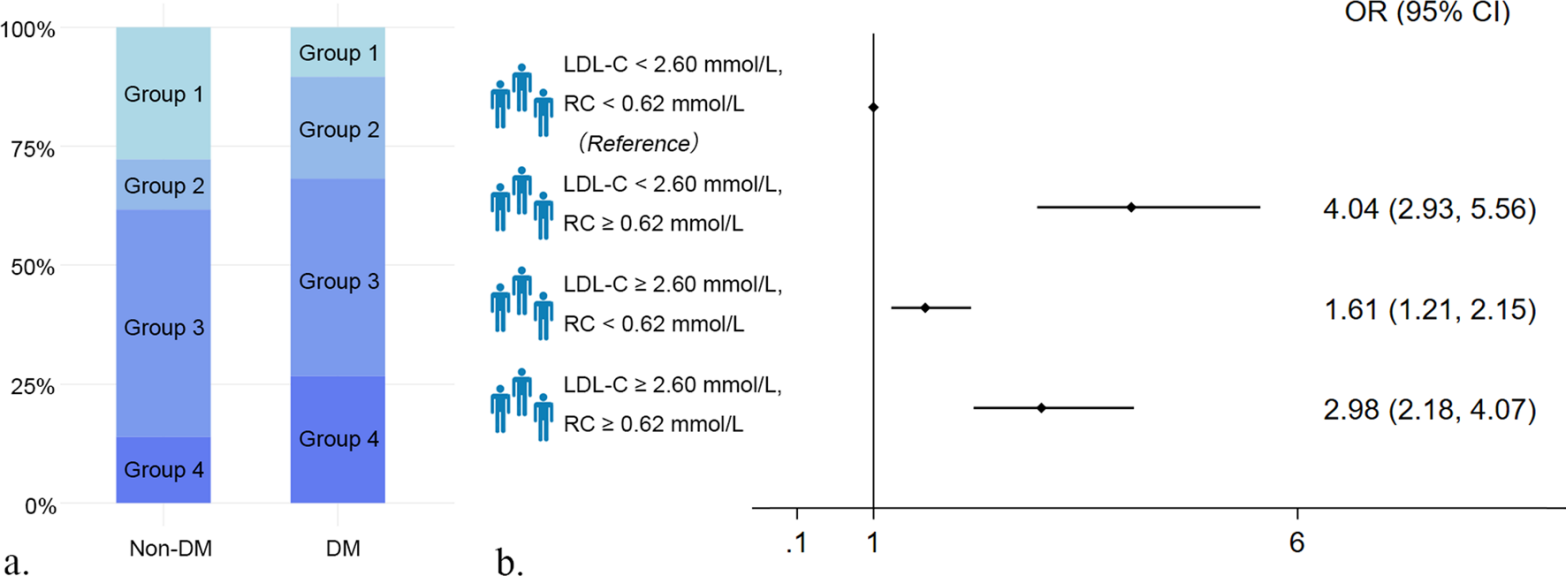
Association of the discordance/concordance of LDL-C (2.60 mmol/L cutoffs) and RC (0.62 mmol/L cutoffs) with DM. **a** The proportion of the discordant/concordant groups in DM and non-DM population. **b** The odds ratios (95% CIs) of DM according to the discordance/concordance of LDL-C and RC. Model adjusted for age, sex, BMI, educational level, smoking, alcohol consumption, and chronic kidney disease. *DM* diabetes mellitus, *LDL-C* low-density lipoprotein cholesterol, *RC* remnant cholesterol, *OR* odds ratio, *CI* confidence interval

Univariate analysis showed that age, BMI, educational level, smoking, alcohol consumption, CKD, the discordant/concordant LDL-C and RC, elevated WBC count and hs-CRP were all significantly associated with DM (Additional file 1: Table S1). Using the multivariate adjusted model for age, sex, BMI, educational level, smoking, alcohol consumption and CKD, we observed that participants in Group 2, Group 3 and Group 4 were associated with 304%, 61%, and 198%, respectively, increased risk for DM (OR: 4.04, 95% CI 2.93–5.56 for Group 2; OR: 1.61, 95% CI 1.21–2.15 for Group 3; and OR: 2.98, 95% CI 2.18–4.07 for Group 4) relative to Group 1 (Fig. 2). Other models in which we considered TG, anti-hypertensive drugs and LDL-C/Apo-B ratio in the analysis also revealed that Group 2 and Group 4 still related with the increased risk for DM (Additional file 1: Table S2).

### Subgroup and sensitivity analysis

In the subgroup analysis, the test for interactions was significant for sex (p for interactions < 0.05), while we noted no significant interactions for age (Additional file 1: Fig. S2). Sex modified the effect of the discordant/concordant LDL-C and RC on DM, with a low LDL-C/high RC ratio more likely to be related to DM in females. We evaluated the association between the discordant/concordant LDL-C and RC groups and DM using stricter clinical LDL-C cut-points (1.80 mmol/L). In this sensitivity analysis, after adjusting the variables in Model 3, we found that a low LDL-C/high RC was still the one most closely related to DM (Additional file 1: Fig. S3).

### Mediator analysis

**Fig. 3:**
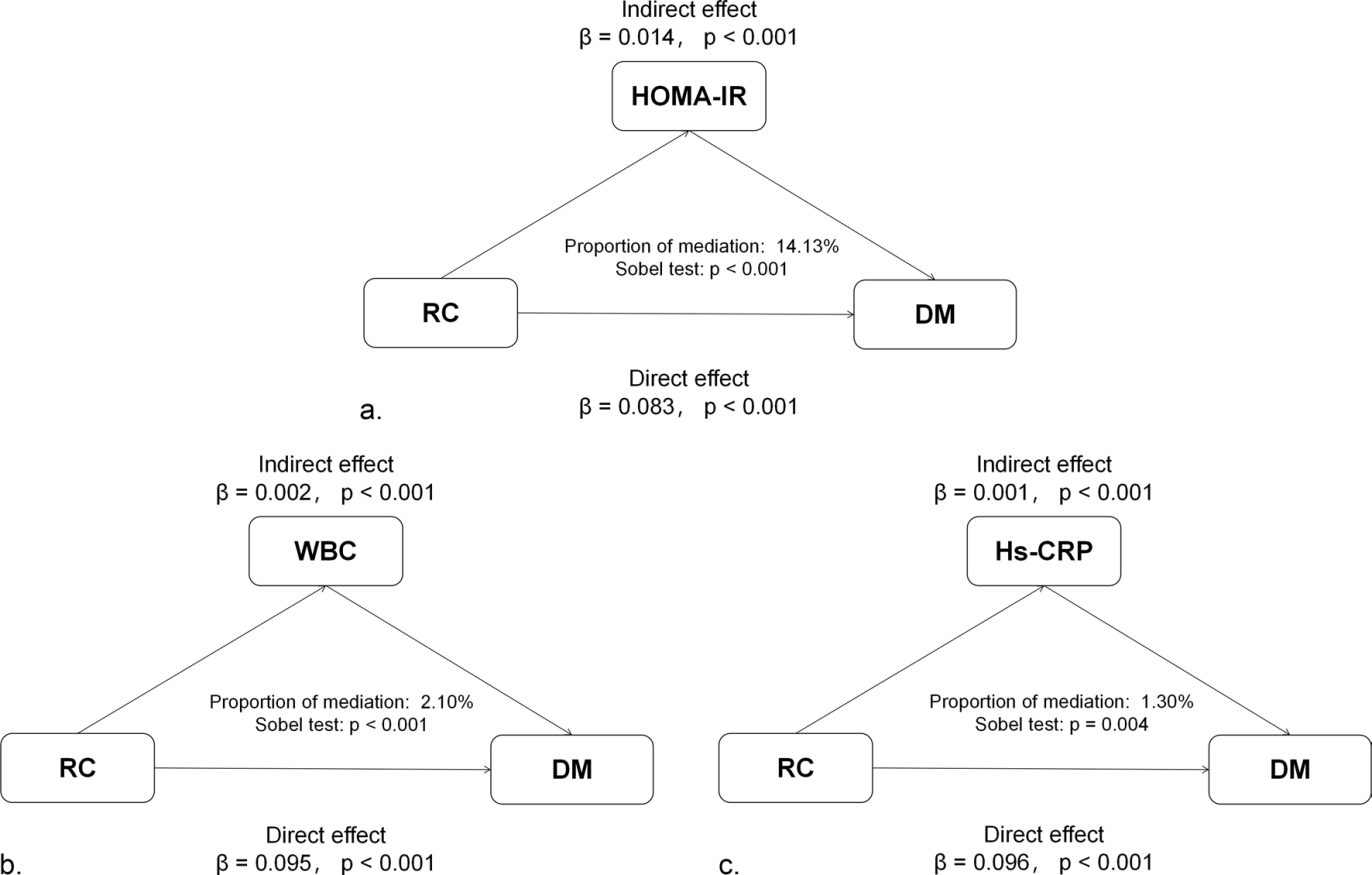
Mediation analysis of the association between RC and DM.  *RC* remnant cholesterol, *DM* diabetes mellitus, *HOMA-IR* homeostasis model assessment of insulin resistance, *Hs-CRP* high-sensitivity C-reactive protein, *WBC* white blood cell

When we assessed the potential mediation effects in the correlation between RC and DM, we noted that HOMA-IR, WBCs, and hs-CRP were significant mediators of the relationship between RC and DM using the Sobel test. We observed significant indirect effects of HOMA-IR, WBCs, and hs-CRP between RC and the risk of DM with the three indices mediated at 14.13%, 2.10%, and 1.30% of the association, respectively (Fig. 3).

## Discussion

We herein found that the discordant/concordant LDL-C and RC were associated with DM to varying degrees. Among them, the low LDL-C/high RC, but not high LDL-C/low RC, was highly associated with DM, suggesting the role of RC beyond LDL-C in DM among the general population. More importantly, this relationship was stronger in females. In addition, the mediation of insulin resistance and the pro-inflammatory state may be the reason why RC is more closely related to DM. Early recognition of the discordant/concordant LDL-C and RC can help distinguish high-risk patients who are predisposed to DM.

As TRLs are cholesterol-rich, RC has attracted attention for its pathogenic capability due to a recently proposed concept—that of cholesterol toxicity—and this has engendered novel perspectives on the development of DM [34]. Previous cohort studies of the general population and coronary artery disease patients suggested that RC was a predictor of new-onset hyperglycemia, superior to other traditional lipid parameters [17, 18, 35]. Another study conducted in a kidney transplant cohort also demonstrated a significant association of baseline RC with new onset diabetes after transplantation [36].

Our results confirmed the previous studies in which elevated LDL-C and RC were implicated in the development of DM. Importantly, we further clarified the role of RC beyond that of LDL-C in the contribution of cholesterol to DM, expanding our knowledge of the pathogenesis of RC. As two large primary prevention clinical studies indicated that RC, but not LDL-C, was associated with the morbidity of atherosclerotic cardiovascular disease [21, 37], the adverse effects of RC may thus be far greater than those with LDL-C. However, there are currently fewer studies that have focused on the relationship between the LDL-C and RC discordance/concordance and DM in the general population. Our result indicated that discordance in LDL-C and RC could be a surrogate for DM, which is a beneficial supplement to the development of DM. Moreover, we have also demonstrated that females are more sensitive to DM from RC exposure, which was consistent with a previous study [18]. Currently, sex differences in discordance/concordance in LDL-C and RC have not been well studied, and we consider that this difference may be related to different dietary structures and cholesterol metabolism. It’s worth noting that women require stricter RC management.

Although the mechanisms underlying the RC-induced disturbance of blood glucose metabolism are not fully understood, the damage of cholesterol overload to β-cells is well documented. Animal experiments have shown that a high-cholesterol environment inhibits the survival of pancreatic β-cells, resulting in the inhibition of insulin secretion. However, cholesterol-lowering strategies benefit pancreatic β-cell function [38–41]. Excess cholesterol is also able to induce endoplasmic reticulum stress and mitochondrial dysfunction and promote reactive oxygen species (ROS) production in pancreatic β-cells, ultimately leading to structural changes in insulin-containing granules [34]. RC is more abundant, larger, and carries more cholesterol than LDL-C particles; hence, it can be inferred that RC is more harmful to pancreatic β-cells [8]. Furthermore, unlike LDL-C, the unique pro-inflammatory characteristics of RC may significantly contribute to abnormal glucose metabolism by exacerbating insulin resistance and a systemic pro-inflammatory state [20, 42]. Apart from these effects, the particle size of TRLs has drawn great interest from researchers, and an increasing body of evidence suggests that the particle size of TRLs is the key factor that contributes to its pathogenicity [8, 43]. Specific particle properties of TRLs and LDL-C are associated with β-cell function and incident DM [8].

There is currently no clear evidence that supports a critical role for insulin resistance and pro-inflammation in the relationship between RC and DM. Ohnishi et al. and Funada et al. previously reported a correlation between RC and insulin resistance [44, 45]. Modern lipoprotein subclass studies have also depicted particle sizes and concentrations of VLDL and LDL-C (as the principal components of RC) as being associated with insulin resistance in a prediabetic population [3, 4, 46]. Insulin resistance, then, also affects RC metabolism. Insulin resistance in the liver can impair the translocation of LRP1 from intracellular vesicles to the hepatocyte plasma membrane, and this then leads to a reduced clearance of TRLs [47]. In addition, an attenuated clearance of TRLs by hepatocytes results in an accumulation of RC [48]. These results may explain the role of insulin resistance in mediating the relationship between RC and DM. In addition, numerous studies have revealed that increased inflammatory-cell infiltration of pancreatic islets in type 2 DM is generally associated with the degree of β-cell dysfunction during the development of diabetes [49]. Islet-resident macrophages and islet-cell inflammation induced by the overproduction of cytokines and ROS are components of the etiology and pathogenesis of human type 2 DM [31, 50]. In our study, we observed that elevated RC was associated with increased WBCs and hs-CRP, suggesting that high levels of RC were often accompanied by a pro-inflammatory state. The mediation analyses showed that the WBC count and CRP concentrations were mediators of the relationship between RC and DM, but that each aspect’s mediating effect was relatively weak. Therefore, we posit that the systemic pro-inflammatory response caused by elevated RC may be involved in the onset and development of DM.

The relationship between RC and DM may also be regulated by mechanisms of RC clearance. For example, peripheral lipoprotein lipase (LPL) and adiponectin are associated with the metabolism of TRLs and involved in the clearance of RC [51, 52]. Shirakawa et al. found that the residual-lipoprotein particle size (which is regulated by circulating LPL and adiponectin) was significantly larger in type 2 DM patients than that in healthy individuals [53]. This finding implicated a potential role for RC accumulation in DM due to inactive LPL and adiponectin, and this might explain the closer relationship between RC and DM from another perspective.

### Limitations

We acknowledge several limitations of the present study. First, we could not draw causal conclusions regarding the pathogenesis of RC and DM due to the cross-sectional nature of the study design. Second, limited by the data provided by CHNS, information on previous statin use could not be obtained. Therefore, the effect of statin use on the relationship between the discordant/concordant LDL-C and RC and DM requires further investigation. In addition, the particle size of the TRLs were not available for more precise classification and assessment of RC. Third, although we executed our analyses via different methods to support our findings, caution must be exercised in interpreting these results as some observed associations may have been incidental. Fourth, RC includes the cholesterol component of VLDL and IDL in the fasting state. However, in the non-fasting blood sample, RC would contain the additional cholesterol component of chylomicron remnants than fasting state. The contribution of RC to DM beyond LDL-C in the non-fasting state needs to be clarified in future research. Finally, the mediation analysis assumed a certain sequence of effects, and our cross-sectional study design limited our directional determination of these effects, thus serving as a potential limitation that is encountered in cross-sectional studies in general.

## Conclusions

The present study provided evidence for the association between the discordance/concordance in LDL-C and RC levels and DM in the general population, and this relationship was partially mediated by insulin resistance and the pro-inflammatory state. In addition, females are more sensitive to DM associated with elevated RC. These findings have important clinical implications for the pathogenesis underlying the RC-induced impairment of glucose metabolism.

## Supplementary Information

**Additional file 1: Table S1.** Univariate and multivariate analysis for DM.**TableS2.** The correlation between discordant/concordant LDL-C and RC and DM. **Figure S1.** Study flowchart. **Figure S2.**Subgroup analyses stratified by patient characteristics. **Figure S3.** Associationof discordance/concordance of LDL-C (1.80 mmol/L cutoffs) and RC (0.62 mmol/Lcutoffs) with DM.

## Data Availability

The data analyzed in this study can be available at: https://www.cpc.unc.edu/projects/china.

## Abbreviations

apoA: Apolipoprotein A
apoB: Apolipoprotein B
BMI: Body mass index
CHNS: China health and nutrition survey
CI: Confidence interval
CKD: Chronic kidney disease
DM: Diabetes mellitus
eGFR: Estimated glomerular filtration rate
FBG: Fasting blood glucose
HbA1c: Glycosylated hemoglobin A1c
HDL-C: High-density lipoprotein cholesterol
HOMA-IR: Homeostasis model assessment of insulin resistance
HOMA-IS: Homeostasis model assessment of insulin sensitivity
Hs-CRP: High-sensitivity C-reactive protein
LDL-C: Low-density lipoprotein cholesterol
LPL: Lipoprotein lipase
OR: Odds ratio
RC: Remnant cholesterol
ROS: Reactive oxygen species
TC: Total cholesterol
TG: Triglyceride
TRLs: Triglyceride-rich lipoproteins
VLDL: Very low-density lipoproteins
IDL: Intermediate-density lipoproteins
WBC: White blood cell
