# Supplementary material for: The role of remnant cholesterol beyond low-density lipoprotein cholesterol in diabetes mellitus
Source: Cardiovasc Diabetol. 2022 Jun 27;21:117. doi: 10.1186/s12933-022-01554-0 (PMC9238255; doi:10.1186/s12933-022-01554-0)
Supplement: Supplementary file 1 — Additional file 1: Table S1. Univariate and multivariate analysis for DM.TableS2. The correlation between discordant/concordant LDL-C and RC and DM. Figure S1. Study flowchart. Figure S2.Subgroup analyses stratified by patient characteristics. Figure S3. Associationof discordance/concordance of LDL-C (1.80 mmol/L cutoffs) and RC (0.62 mmol/Lcutoffs) with DM. [file 12933_2022_1554_MOESM1_ESM.docx]

The role of remnant cholesterol beyond low-density lipoprotein cholesterol in diabetes mellitus

Additional file 1

Table S1. Univariate and multivariate analysis for DM

Table S2. The correlation between discordant/concordant LDL-C and RC and DM

Figure S1. Study flowchart

Figure S2. Subgroup analyses stratified by patient characteristics

Figure S3. Association of discordance/concordance of LDL-C (1.80 mmol/L cutoffs) and RC (0.62 mmol/L cutoffs) with DM

Table S1. Univariate and multivariate analysis for DM

|  | Univariate | | | Multivariate | | |
| --- | --- | --- | --- | --- | --- | --- |
|  | OR | 95% CI | p-value | OR | 95% CI | p-value |
| Age, year | 1.05 | 1.05–1.06 | <0.001 | 1.04 | 1.03–1.05 | <0.001 |
| Male sex | 0.85 | 0.72–1.01 | 0.068 | 0.85 | 0.67–1.08 | 0.191 |
| BMI, kg/m^2^ | 1.23 | 1.20–1.26 | <0.001 | 1.11 | 1.08–1.13 | <0.001 |
| Junior high school or above | 0.51 | 0.43–0.61 | <0.001 | 0.90 | 0.74–1.10 | 0.307 |
| Smoking | 1.25 | 1.05–1.49 | 0.014 | 0.92 | 0.72–1.17 | 0.486 |
| Alcohol consumption | 1.56 | 1.24–1.97 | <0.001 | 1.15 | 0.89–1.49 | 0.288 |
| Chronic kidney disease | 1.91 | 1.60–2.26 | <0.001 | 0.97 | 0.75–1.25 | 0.815 |
| WBCs, 10^9^/L | 1.15 | 1.10–1.20 | <0.001 | 1.08 | 1.04–1.12 | <0.001 |
| TG, mmol/L | 1.42 | 1.36–1.48 | <0.001 | 1.34 | 1.27–1.42 | <0.001 |
| Elevated Hs-CRP | 3.33 | 2.68–4.13 | <0.001 | 1.73 | 1.39–2.14 | <0.001 |
| Discordant LDL-C and RC |  |  |  |  |  |  |
| Low LDL-C/low RC | Reference | - | - | Reference | - | - |
| Low LDL-C/high RC | 5.41 | 3.97 - 7.37 | <0.001 | 1.55 | 1.06–2.27 | 0.025 |
| High LDL-C/low RC | 2.32 | 1.76–3.06 | <0.001 | 1.47 | 1.10–1.96 | 0.009 |
| High LDL-C/high RC | 5.12 | 3.80–6.89 | <0.001 | 1.63 | 1.17–2.28 | 0.004 |

*OR* Odds ratio, *CI* Confidence interval, *BMI* Body mass index, *WBC* White blood cell, *TG* Triglyceride, *Hs-CRP* High sensitivity C-reactive protein, *LDL-C* Low-density lipoprotein cholesterol, *RC* Remnant cholesterol,

Table S2. The correlation between discordant/concordant LDL-C and RC and DM

|  | Model 1 | Model 2 | Model 3 | Model 4 | Model 5 |
| --- | --- | --- | --- | --- | --- |
| Discordant LDL-C and RC |  |  |  |  |  |
| Low LDL-C/low RC | Reference | Reference | Reference | Reference | Reference |
| Low LDL-C/high RC | 5.41 (3.97 - 7.37) | 4.06 (2.95 - 5.59) | 4.04 (2.93 - 5.56) | 1.62 (1.11 - 2.36) | 2.20 (1.53 - 3.16) |
| High LDL-C/low RC | 2.32 (1.76 - 3.06) | 1.60 (1.21 - 2.13) | 1.61 (1.21 - 2.15) | 1.47 (1.11 - 1.97) | 1.92 (1.44 - 2.57) |
| High LDL-C/high RC | 5.12 (3.80 - 6.89) | 2.94 (2.15 - 4.01) | 2.98 (2.18 - 4.07) | 1.73 (1.24-2.40) | 2.56 (1.87 - 3.50) |
| p for trend | < 0.001 | < 0.001 | < 0.001 | 0.005 | < 0.001 |

Model 1: unadjusted;

Model 2: adjusted for age, sex, BMI;

Model 3: adjusted for variables in Model 2, educational level, smoking, alcohol consumption and CKD;

Model 4: adjusted for variables in Model 3, TG.

Model 5: adjusted for variables in Model 3, LDL-C/ApoB, previous anti-hypertensive drug use.

*DM* Diabetes mellitus, *LDL-C* Low-density lipoprotein cholesterol, *RC* Remnant cholesterol

Figure S1. Study flowchart


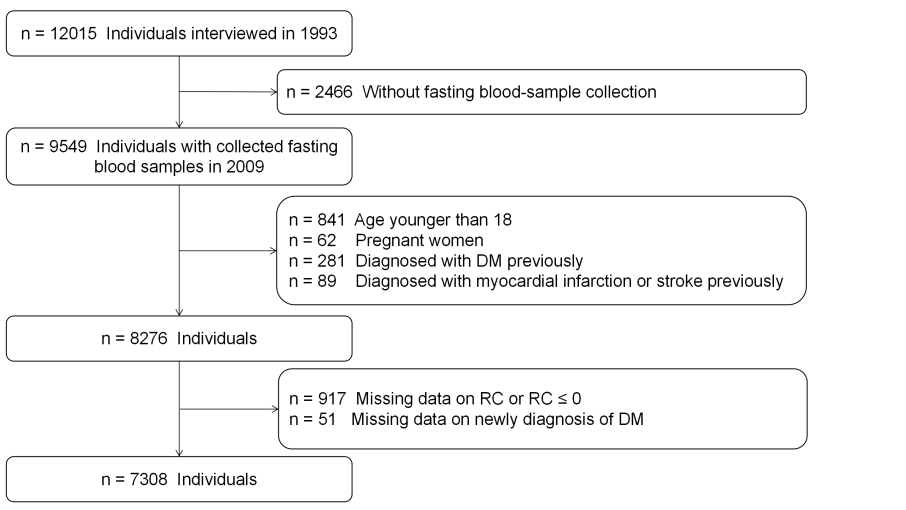


*DM* Diabetes mellitus, *RC* Remnant cholesterol

Figure S2. Subgroup analyses stratified by patient characteristics


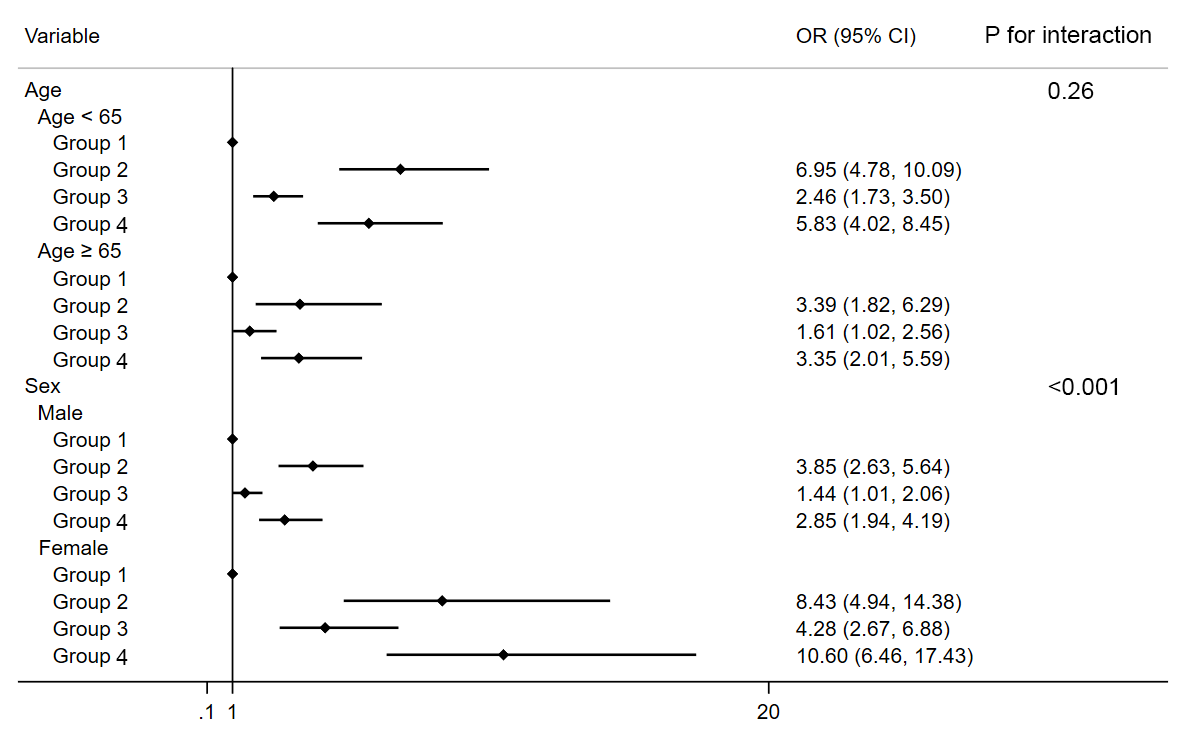


*OR* odds ratio, *CI* Confidence interval

Figure S3. Association of discordance/concordance of LDL-C (1.80 mmol/L cutoffs) and RC (0.62 mmol/L cutoffs) with DM


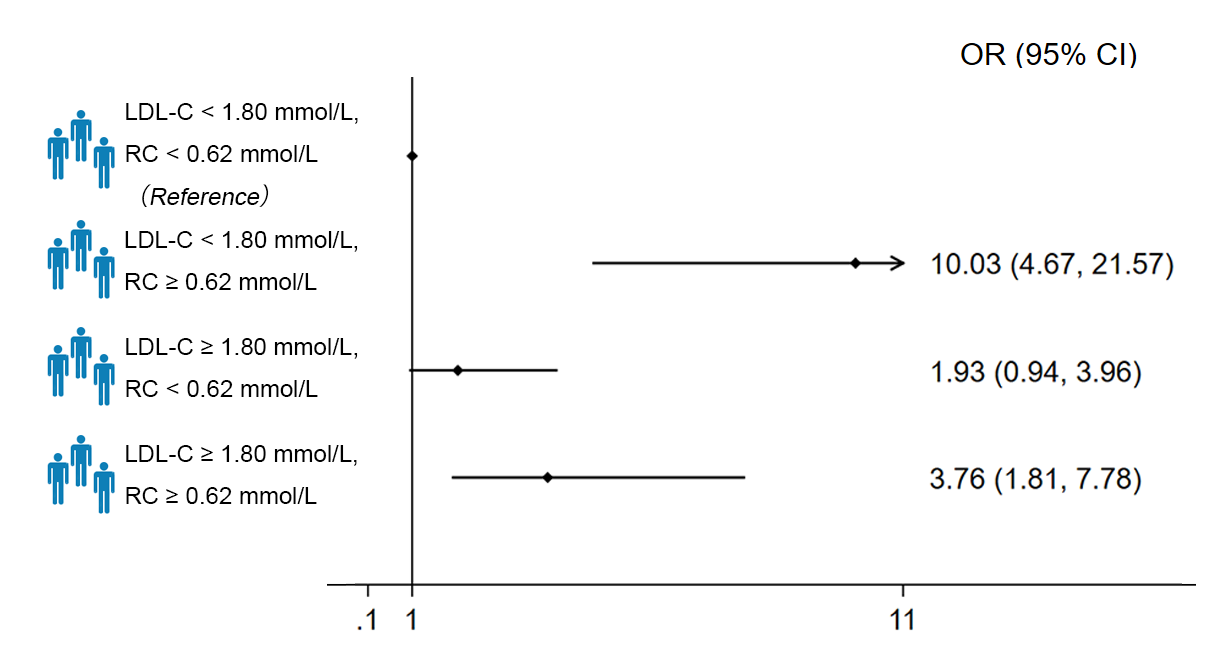


*OR* odds ratio, *CI* Confidence interval, *LDL-C* Low-density lipoprotein cholesterol, *RC* Remnant cholesterol
